# The Presentation, Clinical Diagnosis, Risk Factors, and Management of Rapidly Progressive Hip Osteoarthritis: A Narrative Literature Review

**DOI:** 10.3390/jcm13206194

**Published:** 2024-10-17

**Authors:** Andrei Oprișan, Andrei Marian Feier, Sandor-Gyorgy Zuh, Octav Marius Russu, Tudor Sorin Pop

**Affiliations:** 1 Doctoral School, George Emil Palade University of Medicine, Pharmacy, Science, and Technology of Targu Mures, 540142 Targu Mures, Romania; andrei.oprisan@umfst.ro; 2 Department of Orthopaedics and Traumatology, Clinical County Hospital of Mureș, 540139 Targu Mures, Romania; sandor.zuh@umfst.ro (S.-G.Z.); octav.russu@umfst.ro (O.M.R.); tudor.pop@umfst.ro (T.S.P.); 3 Department M4 Clinical Sciences, Orthopedics and Traumatology I, George Emil Palade University of Medicine, Pharmacy, Science, and Technology of Targu Mures, 540139 Targu Mures, Romania

**Keywords:** rapidly progressive hip osteoarthritis, clinical diagnosis in rapidly destructive hip osteoarthritis, rapidly progressive coxopathy, rapidly destructive coxopathy, clinical presentation, clinical management

## Abstract

Rapidly progressive hip osteoarthritis (RPOH) is a rare and severe form of osteoarthritis (OA), marked by the rapid degeneration and destruction of the femoral head, often within months. Despite its unclear etiology, several factors such as subchondral fractures and immune responses have been proposed as possible contributors. This narrative review aims to synthesize current knowledge on the pathogenesis, risk factors, clinical presentation, imaging features, and grading systems of RPOH. Predominantly affecting elderly females, RPOH presents distinctive challenges in both diagnosis and management due to its abrupt onset and severity. Known risk factors include advanced age, female gender, obesity, intra-articular corticosteroids use, and long-term hemodialysis. Clinically, RPOH is characterized by severe pain during active weight-bearing movements, despite patients presenting a normal range of motion during passive examination in the early stages. While several classification systems exist, there is no universal standard, complicating differential diagnosis and clinical approaches. This review emphasizes the necessity for early diagnostic methods utilizing specific biomarkers, rapid differential diagnosis, and targeted, personalized interventions based on individual risk factors.

## 1. Introduction

Rapidly progressive hip osteoarthritis (RPOH) is a rare yet debilitating condition that presents significant clinical and diagnostic challenges. Every year, countless patients experience the devastating effects of RPOH, a condition that can severely destruct a fully functional hip joint in a few months. Despite its rarity, the aggressive nature of RPOH leads to sudden disabilities, impacting both patients and healthcare providers. Since its initial description by Forestier in 1957 [1], RPOH has confused clinicians with its enigmatic pathogenesis and rapid clinical course. The condition was later termed “rapidly destructive hip osteoarthritis” by Lequesne and Postel in the 1970s [2,3], and the nomenclature for this condition has undergone several evolutions over time, being described in the literature in various forms such as “rapid destructive coxarthrosis”, “atrophic osteoarthritis”, “rapidly progressive osteoarthritis”, and “Postel’s osteoarthritis”. RPOH is characterized by a swift destruction of the femoral head and acetabular bone stock, often leading to the complete disappearance of the femoral head within a few weeks or months [4,5]. The etiology of RPOH remains largely enigmatic, with hypothesized causes ranging from drug toxicity and subchondral osteonecrosis to ischemia and cytokine-mediated immunological mechanisms [3]. This obscure pathogenesis underscores the need for a comprehensive review of the existing literature to better understand the condition’s underlying mechanisms.

Furthermore, the clinical presentation of RPOH, primarily affecting elderly females, often with unilateral involvement, implies severe hip pain with a partially preserved range of motion necessitating total hip replacement [4,5]. Recent case reports highlight not only the swiftness of joint deterioration but also the lack of consensus on diagnostic criteria and management strategies. With incidence rates of rapid progression in patients ranging from 5% to 20% [6], there is an urgent need to consolidate the existing knowledge. The categorization of RPOH into three types based on the pattern and rate of joint destruction further complicates its diagnosis and treatment, necessitating a detailed review of the clinical characteristics and therapeutic approaches [6].

In recent years, a number of case series have been reported, yet a consolidated synthesis of this information is lacking. With this gap in understanding, this review aims to synthesize the current understanding of RPOH pathophysiological mechanisms and risk factors and evaluate the clinical and imaging features to improve early detection, personalize treatment approaches, and outline further required research.

## 2. Methods

We conducted a narrative review to synthesize the current knowledge and state of the art on RPOH. The analysis focused on identifying key studies that explored the pathogenesis, risk factors, clinical presentation, imaging features, and classification/grading system for RPOH.

### 2.1. Literature Search Strategy

A comprehensive literature search was performed using PubMed, Scopus, and Cochrane databases up to 30 March 2024. The search terms included “rapidly progressive hip osteoarthritis”, “rapidly destructive hip osteoarthritis”, “rapid coxarthrosis”, “rapidly progressive osteoarthritis”, “hip osteoarthritis”, “femoral head destruction”, and “RPOH risk factors”. The search was limited to articles published in English, and no timeframe restrictions were applied.

### 2.2. Data Extraction and Synthesis

Two independent reviewers screened the titles and abstracts of the identified articles. Full texts of potentially relevant studies were retrieved and assessed for eligibility. Key data extracted from each study included the study design, patient demographics, diagnostic criteria used, classifications, clinical outcomes, and any proposed mechanisms of disease progression. Data were synthesized narratively due to the heterogeneity in study designs and outcome measures. The synthesis included a qualitative assessment of the evidence on the pathogenesis, clinical features, risk factors, and grading systems used. Where applicable, emerging trends and gaps in the literature were highlighted.

## 3. Pathogenesis

RPOH is characterized by an accelerated bone destruction process, reinforced by unique cellular and molecular pathways [7]. These pathways potentially drive the accelerated degeneration of the femoral head. Current research has not fully elucidated the underlying causes of rapid progression. An increased number of osteoclasts has been implicated as a contributing factor in the pathogenesis [8]. This phenomenon is characterized by an elevated presence of osteoclasts not only in the affected regions of the femoral head but also within the synovium [9]. Yokota et al. ubiquitously identified osteoclasts in the synovial tissues of patients diagnosed with RPOH, a distinct observation contrasting with their absence in the synovial tissues of patients suffering from idiopathic degenerative osteoarthritis (OA) or osteonecrosis of the femoral head (ONFH) [10]. The differential presence of osteoclasts across these patient groups may suggest a unique pathological mechanism underlying RPOH, distinct from that of OA and ONFH. Historically, an augmented vascularity in the femoral head, coupled with a histologically observed increase in macrophage density, has been documented as a pathological characteristic in the progression of the disease [11]. The role of existing inflammasomes may be intricately linked to inflammatory responses and promote synovitis along with osteoclast differentiation and osteoclastogenesis within the joint, thereby underlying a distinctive pathophysiological pathway of RPOH [10,12]. Elevated inflammasome expression in synoviocytes has also been observed, potentially exacerbating quick degradation of joint components.

**Figure 1:**
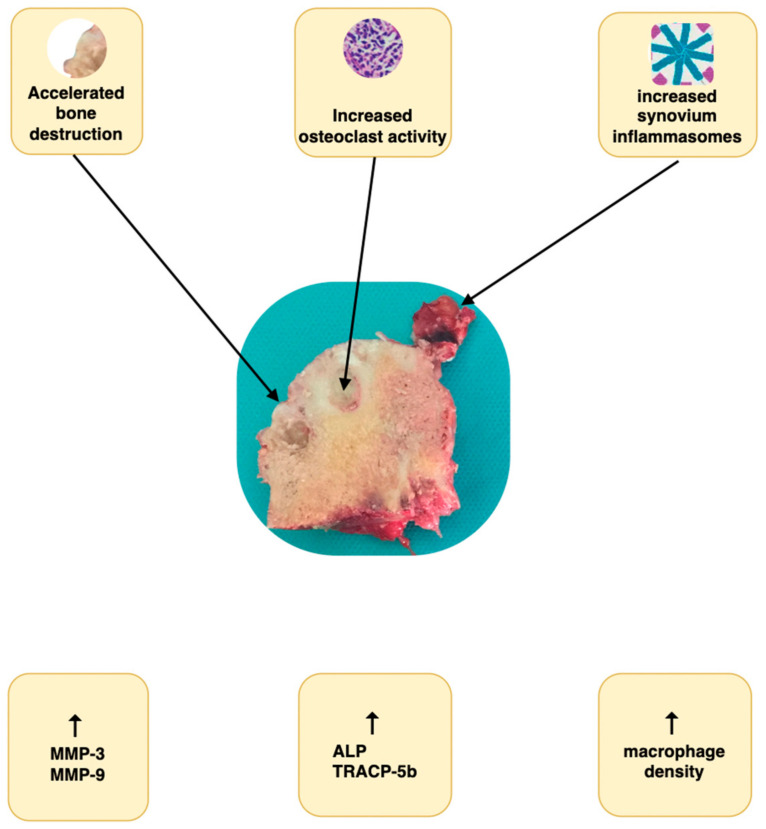
Known cellular and molecular mechanisms in the pathogenesis of RPOH (macroscopic specimen from femoral head section: personal archive) [14,18,19].

Matrix metalloproteinases-3 and -9 (MMP-3 and MMP-9) have been implicated in the rapid degradation of cartilage and subsequent bone loss [13,14,15] with their exact mechanism unknown to date. In a comprehensive comparative analysis of biomarker activity between RPOH and OA, Abe et al. elucidated that markers indicative of cartilage destruction are significantly elevated in RPOH [16]. Additionally, they observed a notable increase in bone turnover markers, such as alkaline phosphatase and tartrate-resistant acid phosphatase isoform 5b (TRACP-5b), in RPOH compared to idiopathic OA. This finding further confirms the hypothesis of extensive osteoclast activation occurring concurrently in both synovial fluid and bone tissue, reinforcing the underlying mechanisms proposed in the pathogenesis (Figure 1).

Several researchers have explored the potential involvement of pelvic angles, along with alterations in the center-edge angle and anterior acetabular head index, as contributing factors to the etiology of RPOH [16]. Nonetheless, this association remains a subject of contention within the scientific community, with conflicting results reported in other studies [11,17].

## 4. Risk Factors

A significant overlap emerges with conditions prevalent in both the general elderly population and individuals diagnosed with idiopathic OA. This intersection, particularly in the context of associated comorbidities and family history, poses a subtle challenge in delineating risk factors that are distinctly characteristic of RPOH, as opposed to those common in broader demographic and disease categories. A classification of identified risk factors is structured in Table 1.

A potential critical factor implicated in the progression of RPOH is the regular use of non-steroidal anti-inflammatory drugs (NSAIDs). The incidence of NSAID use was notably higher in patients with RPOA compared to those with normally progressive hip OA [6]. Theories suggest that NSAIDs might contribute through mechanisms such as direct drug toxicity or by providing analgesic relief that leads to increased joint stress and subsequent destruction, a phenomenon sometimes referred to as “analgesic hip” [11].

Emerging evidence emphasizes the significance of advanced age as a risk factor in the development of RPOH. Studies have consistently reported a higher mean age among patients with RPOH compared to controls. RPOH predominantly affects female elderly individuals, typically presenting between 60 and 70 years of age [23]. This correlation may be attributed to age related biological changes, such as modified bone density and altered cartilage composition, which increase susceptibility to wear-and-tear cartilage damage and subchondral insufficiency fractures [17,24]. Diagnostic processes in older patients may be complicated by the attribution of hip pain and mobility issues to general aging, delaying the early diagnosis and identification of an aggressive form of OA [25]. A retrospective analysis revealed that the mean age for RPOH patients was 72 years [26]. Moreover, older patients demonstrated a considerable degree of acetabular bone loss (averaging 10.0 mm at 12 months), underscoring the severity of the condition and treatment challenges in the aging demographic.

A gender predisposition is observed, showing that females are at significantly higher risk for developing RPOH. This gender-related vulnerability is repeatedly reported [11,17,26], with a notable majority of the affected population being women, presumably related to post-menopausal hormonal imbalances. In comparative control group studies, there is a methodological trend towards the exclusion of male individuals from cohorts, a practice centered on the recognition that male participants may act as statistical outliers, thereby skewing the population data [8]. Such a methodological decision is reflective of a targeted research strategy aimed at a specific population and to minimize confounding variables and biases. The proportion of female patients in the RPOH group was dominant, in most studies [11,16,17,25], suggesting a strong, if not definitive, gender bias. The observed gender disparity in disease susceptibility underscores a critical need for further targeted research to unravel the underlying biological and socio-environmental factors contributing to this pronounced difference in prevalence between genders.

Long-term hemodialysis, particularly in the context of diabetic nephropathy, obesity, and absence of treatment for inflammatory arthritis or gastrointestinal disorders, has also been reported as a significant risk factor for RPOH [27].

Genetic factors play a significant role in the predisposition and progression of OA, with numerous studies highlighting the impact of specific genes related to collagen structure, inflammatory pathways, and cartilage matrix metabolism in its development. While the genetic foundations of OA are increasingly understood, there remains a notable gap in research specifically addressing RPOH. Consequently, while the insights gained from OA studies offer valuable understanding, direct conclusions about RPOH genetic factors are yet to be drawn and interpreted due to the absence of targeted research in this area.

### 4.1. Modifiable Risk Factors

#### 4.1.1. Obesity and Body Mass Index

Obesity has been increasingly recognized as a potential risk factor in the progression of RPOH. Although the link between obesity and hip OA is less established compared to knee OA, studies suggest that higher body mass index (BMI) may contribute to disease evolution. Data from the Nurses Health Study demonstrated that women with a BMI ≥ 35 kg/m^2^ had a 2.6 times higher risk of requiring total hip replacement compared to those with a BMI under 22 kg/m^2^ [28]. If obesity was present from an early age (BMI ≥ 35 kg/m^2^ at 18 years), the relative risk for hip replacement rose to 5.2, indicating a strong link between long term obesity and severity of hip OA.

Other studies present conflicting evidence, suggesting that BMI does not have a direct impact on the development of RPOH [29]. Instead, other factors such as subchondral fractures or mechanical stress from physical activity after rapid pain relief (e.g., post-injection) may be more significant [30]. This complexity hints that while obesity likely plays a role in accelerating joint damage and progression in some patients, it may not be the sole or primary factor in every case. Further research should focus on well-designed longitudinal cohort studies to explore the long-term impact of obesity and early-life BMI on the progression of RPOH while accounting for other factors such as mechanical stress, subchondral fractures, and genetic predispositions that may influence disease severity.

#### 4.1.2. Intra-Articular Corticosteroids

Intra-articular corticosteroid (ICS) injections have been explored in relation to RPOH with varying opinions on their beneficial and detrimental role. While ICS injections are a common treatment for joint pain, their use in patients with hip OA, particularly those at risk for RPOH, remains controversial [29]. Montero et al. indicated that corticosteroid injections may be implicated in RPOH, as 7% of patients who received injections developed the condition [31]. These patients, however, had other specific predisposing factors, such as advanced age, severe degenerative joint changes, and a narrowed joint space. As a general rule, it is recommended to avoid using such injections in patients with severe coxarthrosis, and doses higher than 40 mg should be avoided, as some studies suggest a higher risk of RPOH with larger doses of 80mg or more [31,32]. Supporting this association, Hess et al. found that 21% of patients who received ICS injections developed RPOH [33]. Interestingly, the study pointed out that the development of RPOH may be more likely in older patients with higher Kellgren Lawrence grades [34], yet it did not find a clear association between the number of injections or BMI. Patients with RPOH were more likely to undergo THA, with 44% of the affected patients requiring surgery within a significantly shorter time compared to those without the rapidly progressive type.

Flemming et al. raised questions about the true causality between ICS injections and RPOH, suggesting that progression might already be underway at the time of injection [30]. Early indicators, such as bone marrow edema on MRI, seem to help predict destructive changes, and in those cases, the ICS injection may simply coincide with the natural course of the disease. On the other hand, Tiwari et al. noted cases of chondrolysis and cartilage damage immediately following ICS use [35]. While systemic steroid use has been associated with avascular necrosis [36], single-shot ICS injections have not been conclusively linked to rapid joint destruction [37], though there remains a concern for potential cartilage damage. Studies suggest a possible link between ICS injections and RPOH, but the evidence is mixed, and the causality remains unclear in the literature. To establish causality, prospective randomized controlled trials are needed, complemented by longitudinal observational studies with detailed imaging that can isolate the effects of ICS from other confounding factors.

### 4.2. Non-Modifiable Risk Factors

#### 4.2.1. Age and Gender

Studies consistently report an increased risk in individuals over 60 years of age, aligning with the general rise in OA incidence with aging [38,39,40,41,42]. Age-related joint degeneration and comorbidities such as rheumatoid arthritis and diabetes mellitus contribute to this increased susceptibility [42]. Women were similarly associated with higher rates of RPHO, particularly in women with autoimmune conditions, with Montero et al. reporting that RPHO has a male-to-female ratio of 1:2 [31,43]. Postmenopausal women experience accelerated joint degradation due to hormonal changes affecting bone density and cartilage health [44]. Some studies report no significant correlation between BMI, BMD, and RPHO development, particularly when adjusted for age and systemic disease [45].

#### 4.2.2. Long-Term Hemodialysis

Multiple studies have highlighted the link between prolonged hemodialysis and the development of hip OA. Nakai et al. observed that patients undergoing long-term hemodialysis (≥10 years) showed marked radiographic changes, such as bone cyst formation and the narrowing of the joint space [46]. Similarly, Fukunishi et al. emphasized that chronic hip arthropathy is a serious complication for those on extended hemodialysis, frequently leading to the need for total hip arthroplasty [47]. Dialysis-related amyloidosis, common in long-term hemodialysis patients, plays a role in the rapid progression of osteoarticular damage in the hip joint, involving the deposition of beta-2 microglobulin [27,48].

#### 4.2.3. Sagittal Spinopelvic Malalignment

Evidence suggests that sagittal spinopelvic alignment has a role in the pathogenesis of hip disorders, including RPOH. Malalignment, mostly increased posterior pelvic tilt, has been identified as a significant contributor to the accelerated degeneration of the hip joint [20]. Degenerative changes in the lumbar spine, such as lumbar kyphosis, are known to exacerbate posterior pelvic tilt, which decreases anterior acetabular coverage and leads to altered load distribution across the femoral head [21,22]. This biomechanical disruption is especially relevant in patients with RPOH, where mechanical stress concentrates in the anterior portion of the femoral head, facilitating accelerated joint destruction. Finite element analyses have demonstrated that posterior pelvic tilt significantly increases stress in the anterosuperior part of the acetabulum. Ojima et al. found that a 20° posterior pelvic tilt increased the mean value of equivalent stress and maximum principal stress in the anterosuperior part by roughly 1.5 times compared to the neutral position [49]. Clinical investigations comparing sagittal spinopelvic alignment between patients with RPOH and those with typical OA have demonstrated distinct differences [50]. RPOH patients exhibit a significant increase in posterior pelvic tilt, reduced lumbar lordosis, diminished lumbar range of motion, and a lower sacral slope when compared to idiopathic hip OA patients. These findings are notable in RPOH grade II where posterior pelvic tilt has been shown to exceed that observed in both developmental dysplasia of the hip and RPOH grade I [51]. The reduction in anterior acetabular coverage resulting from posterior pelvic tilt subsequently increases mechanical loading in the weight bearing zone of the hip, which can accelerate cartilage erosion and subchondral bone destruction. An indicator of femoral bone integrity, cortical thickness index (CTI), has been found to correlate significantly with bone mineral density in the hip [52]. A higher CTI suggests greater bone robustness, yet in RPOH, structural changes in the femur, in combination with pelvic malalignment, may undermine joint stability [53].

Given these biomechanical considerations, adopting spinopelvic alignment measurements in patients suspected of RPOH is crucial for optimizing and predicting treatment outcomes. The mechanical load redistribution caused by posterior pelvic tilt likely exacerbates joint degradation, making pharmacological interventions insufficient to stop disease progression. Corrective measures targeting spinopelvic alignment, such as therapeutic exercises, postural adjustments, or even surgical intervention, should be considered integral components of a complete management strategy for RPOH.

#### 4.2.4. Collagen Structure Gene Implications

Li et al. documented a case study of a family presenting a mutation in the COL2A1 gene, characterized by the nucleotide change c.611G > C, resulting in the amino acid substitution of glycine to alanine at position 204 (Gly204Ala) within the core triple-helical domain of the COL2A1 protein [54]. This specific genetic alteration disrupts the integrity of type II collagen structure, concluding in the phenotypic manifestation of early-onset OA, which affects multiple joints. Moreover, Xia et al. demonstrated that the overexpression of β-catenin leads to severe cartilage degeneration in the femoral head [55]. Cartilage degradation and chondrocyte apoptosis caused by β-catenin activation might be the genetic factors that contribute to early and accelerated progression.

#### 4.2.5. Inflammatory Pathways Implications

Generally, TNF-α and various interleukins have been implicated in the genetic inflammatory mechanisms of OA, with TNF-α specifically inducing the overexpression of retinoic acid receptor gamma (RARγ) and influencing the expression of genes related to matrix degradation and inflammation [56]. The MAPK and TLR pathways, associated with the regulation of OA inflammation, are influenced by IL-1β, IL-6, IL-15, IL-17, IL-18, and RARγ interactions [56,57]. A study on synovial fibroblasts from OA sufferers showed that fibronectin1 was tightly related to the abnormal growth of synovial fibroblasts and could potentially play a key role in early micro-level cartilage alterations [58]. Genes involved in fibroblasts growth, such as those related to extracellular matrix, immune and cell adhesion molecule binding, were significantly activated in cartilage affected by OA changes.

While these factors are well documented in the context of standard idiopathic OA, their potential involvement in the etiology of RPOH remains an area of ongoing research. To date, the direct linkage between these known factors and the RPOH type has not been firmly established by high-evidence-level scientific studies. This gap in knowledge signals the need for further epidemiological and genetic studies to explore whether individuals with RPOH share similar predispositions as those with the standard idiopathic form, discovering shared pathophysiological mechanisms and opening paths for targeted gene and personalized therapeutic strategies.

#### 4.2.6. Cartilage Matrix Metabolism Implications

Several studies have identified various genes associated with cartilage matrix metabolism in hip OA. Notably, a study on bone and cartilage metabolism markers in the synovial fluid of the hip joint found significant differences in the levels of matrix MMP-3 among patients with different hip conditions, including RPOH [59]. The expression levels of MMP-3, which are associated with cartilage metabolism, suggest alterations in cartilage turnover and may implicate these genes in the early onset of pathogenesis. In addition, the increased expression of MMP-1 and MMP-2, along with the reduced expression of TIMP-1, was observed in patients showing early signs of OA compared to normal controls [60]. Furthermore, the aggrecan protein, which is critical for cartilage structural integrity and is coded by the ACAN gene on chromosome 15q26.1, is crucial for providing compressive resistance of the cartilage [61]. Mutations in ACAN can disrupt chondrocyte differentiation and endochondral ossification, contributing to skeletal dysplasias and early-onset OA. While mutations in structural protein genes like ACAN and COL2A1 are linked to skeletal dysplasia and early-onset OA, they are not directly associated with the more common late onset idiopathic OA [62].

Recent findings on inflammasome signaling in RPOH provide more insights into the genetic contributors to hastened disease progression related to matrix metabolism implications and inflammasomes. The inflammasomes role, especially the NLRP3 inflammasome component and the downstream effectors such as Gasdermin D, are required to understand the accelerated joint destruction seen in RPOH, as they lead to the excessive activation of inflammatory pathways and subsequent chondral damage [11,63]. The increased expression of inflammasome components, including the aforementioned upregulation of proinflammatory cytokines IL-1β and TNF-α, as well as enzymes like MMP-3 and MMP-9, emphasizes a potential link between inflammation and cartilage matrix degradation [64]. An altered expression of adhesion modulatory timsin domain containing 4 and 5 (ADMTS4, ADMTS5) and enhanced RANKL expression provide a connection to joint instability and rapid bone destruction, which could be pivotal in the onset of RPOH and may represent therapeutic targets or biomarkers for early detection and novel treatment strategies [65,66,67].

## 5. Presentation and Clinical Features

The clinical features of RPOH beyond pain, range of motion (ROM), stiffness, and lack of crepitus align closely with those observed in idiopathic OA of the hip [26]. This includes functional limitations affecting daily activities such as walking and stair climbing, muscle weakness and atrophy from disuse, gait abnormalities including limping, and significant psychosocial impacts on mental health and social engagement.

### 5.1. Pain

The pain associated with RPOH is marked by its acute severity and rapid onset. Patients typically report a sudden and intense onset of pain, which often occurs in a hip that appears radiologically normal prior to the onset of symptoms [7]. This abrupt and severe presentation is a distinguishing feature of RPOH, differentiating it from more gradually progressive forms of osteoarthritis. It is particularly exacerbated during nocturnal hours, leading to a significant impairment in patient comfort and sleep quality [4]. Within the initial two-month period following diagnosis, the pain intensity is frequently rated between 8 and 10 on the Visual Analogue Scale, often necessitating the use of over-the-counter analgesics [68].

### 5.2. Range of Motion

In the early stages of RPOH, patients typically present with a normal passive ROM during clinical examination, which may obscure the severity of the underlying disease. A feature of RPOH is the excruciating pain experienced during active ROM movements, particularly when weight-bearing. The combination of normal passive ROM and painful active, weight-bearing ROM is a key feature identified in RPOH and sets it apart from other forms of OA and signals the rapid progression of joint destruction [69]. The absence of osteophytes results in a significantly less pronounced limitation in the ROM compared to idiopathic OA [70]. As RPOH advances, pathological changes, including joint space reduction and chondral degradation, manifest, leading to a gradual limitation in the coxo-femoral joint ROM. This restriction predominantly affects movements of internal rotation, abduction, and flexion of the hip [4]. In the most severe manifestations, with an aggressive onset and hastened femoral head destruction, individuals encounter an extreme setting where there is a complete loss of joint movement, highlighting the debilitating impact of the disease at its advanced stage. This progression underscores the critical need for current research in the existing gap of early detection methods and interventions to mitigate the severe functional impairments.

### 5.3. Stiffness

Limited but promising evidence indicates joint stiffness as a significant symptom in RPOH, especially in the morning, likely due to a combination of joint destruction, activation of inflammatory pathways, and disuse muscle hypotrophy [70,71].

## 6. Imaging

The radiologic presentation of RPOH can be dramatic, leading to diagnostic confusion with other arthropathies, infections, and osteonecrosis. The subchondral fracture is believed to play a role in the development of rapid joint destruction and can often be identified on plain AP radiographs.

### 6.1. Radiographs

**Figure 2:**
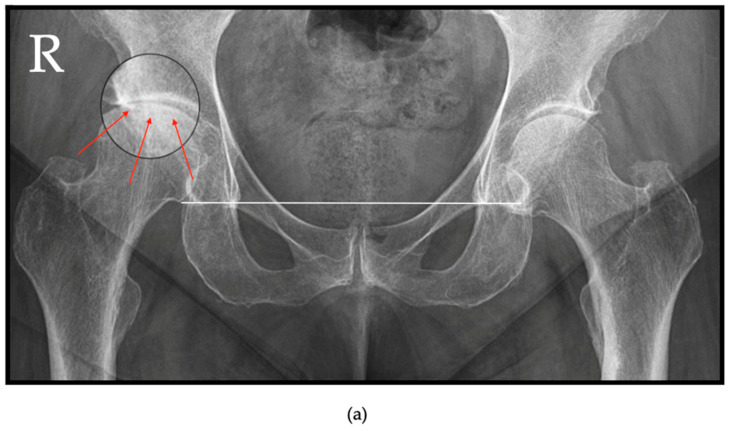

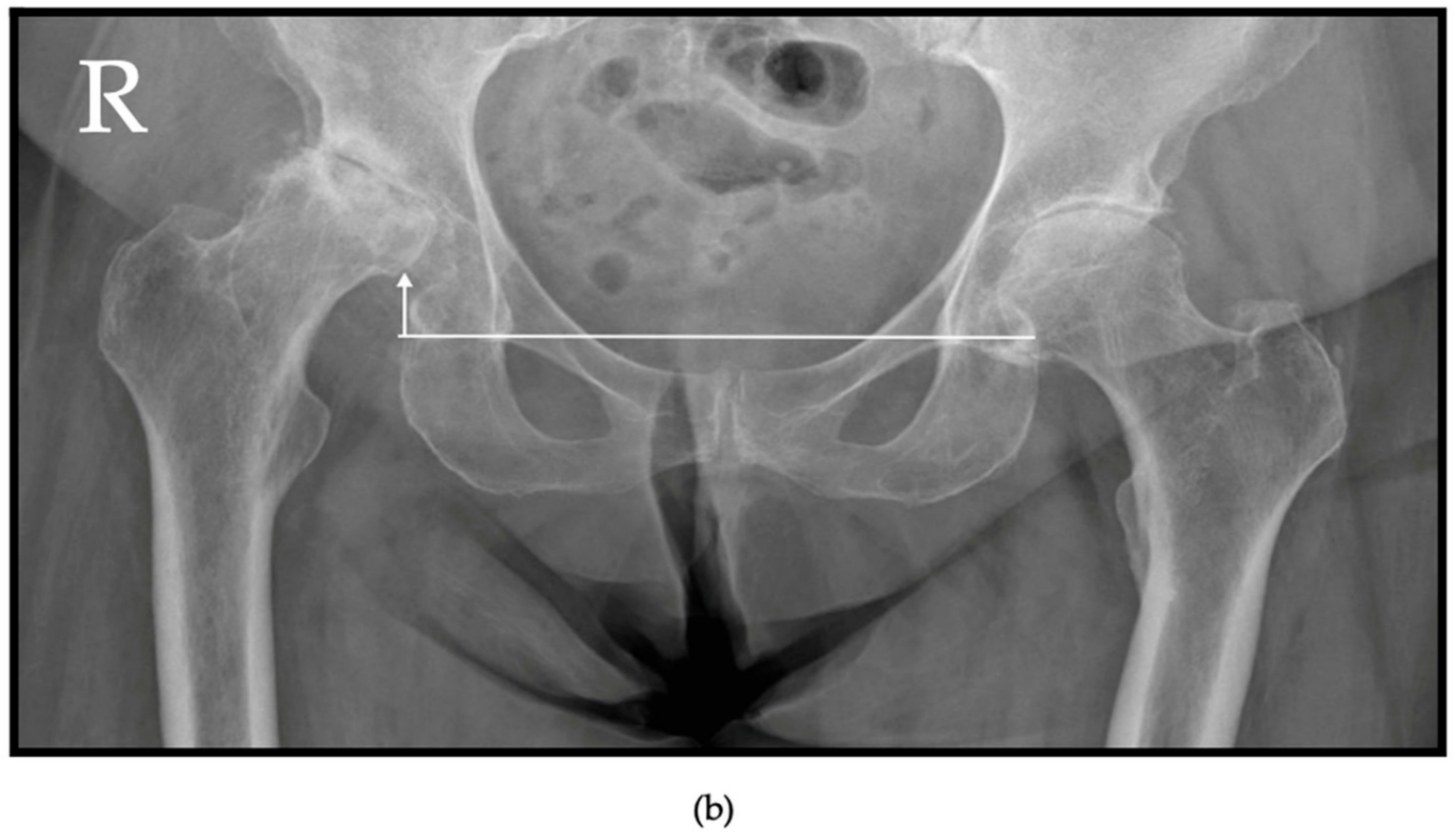
Right RPOH in a 68-year-old female patient—AP views of the pelvis, horizontal white line—connecting the pelvic tear drops; (**a**) subchondral insufficiency fracture located at the weight-bearing area of the right femoral head (magnified area—red arrow) with superior joint space narrowing and no ascension of the femoral head; (**b**) femoral head and acetabular bone resorption with significant ascension of the femoral head. The patient presented with pain during walking in August 2022 (**a**) with a pelvic AP view demonstrating Zazgyva Grade I RPOH; in August 2023 (**b**), a pelvic AP radiographic view demonstrated destruction of the femoral head accompanied by acetabular involvement with elevation of the femoral head exceeding 0.5 cm, corresponding to Zazgyva Grade III, white arrow—ascension of the femoral head from the tear drop line.

RPOH is characterized by two distinct radiographic stages: rapid chondrolysis followed by rapid and marked subchondral bone resorption and destruction [30]. Early chondrolysis stage is marked by minimal joint space narrowing, lack of osteophytes, and scant/minimal subchondral sclerosis [30]. In this stage, conventional radiographs may display either normal anatomical features or subtle degenerative changes. At a more advanced stage (2–6 months), radiographic imaging typically shows a rapid and asymmetric reduction in joint space, particularly in the outer load-bearing regions. Associated findings often include increased subchondral bone density or subchondral insufficiency, presence of subchondral acetabular or femoral head cystic lesions, and minimal or absent osteophyte formation at the superior aspect of the femoral head and the corresponding lateral acetabular region [7]. In some cases, AP pelvis radiographs taken at the onset of pain may reveal the initial subchondral bone fracture (Figure 2a) and subsequent collapse of the femoral head (Figure 2b). This collapse can occasionally extend to involve the acetabulum, resulting in acetabular flattening and eventual complete or partial resorption of the femoral head (Figure 2b).

**Figure 3:**
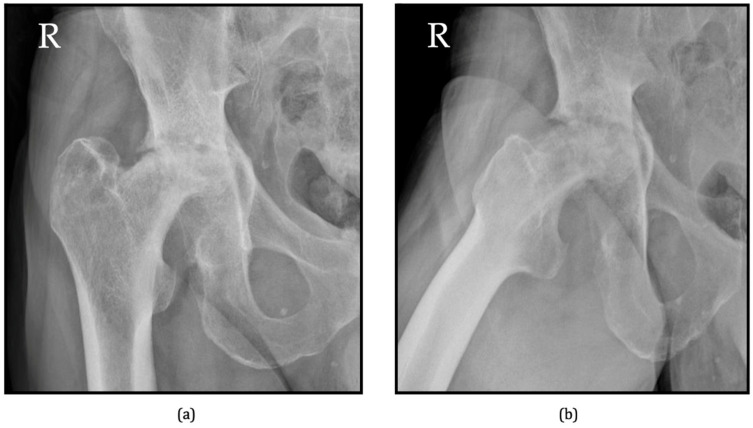

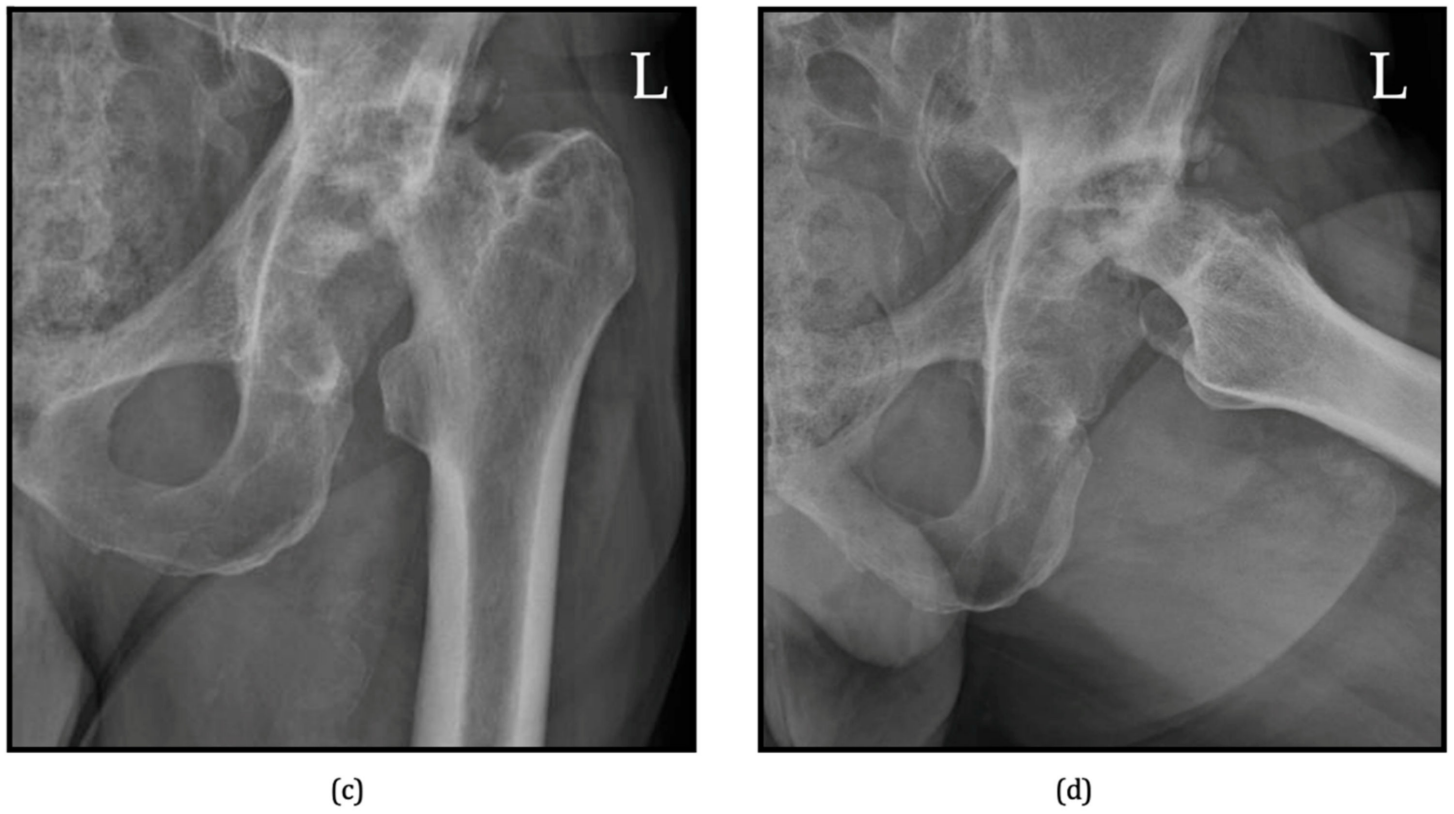
Bilateral RPOH in a 54-year-old male patient—AP views of the coxo-femoral joint ((**a**) right side, (**c**) left side) and axial views of the coxo-femoral joints ((**b**) right side, (**d**) left side). Marked bone resorption can be observed in both hips, involving both the femoral head and the acetabulum (source: personal archive).

Due to the insensitivity of radiographs in the early stages and the potential for misinterpretation with other pathologies such as septic arthritis, inflammatory arthropathies, or avascular necrosis, their utility in early diagnosis can be limited [72]. Bilateral RPOH presentation in male patients is uncommon and considered extremely rare [73]. However, a documented case of bilateral RPHO from our center is presented in Figure 3.

### 6.2. Magnetic Resonance Imaging and Computed Tomography

Given the limitations and overlap of radiographic features with other hip joint disorders in the initial stages, magnetic resonance imaging (MRI) is key for early detection. MRI is capable of identifying early pathological changes that are not apparent on radiographs, such as joint effusion and bone marrow edema (femoral head, neck, and acetabulum) and secondary signs of subchondral insufficiency. A key sign on MRI is a band-like hypointense signal on T1-weighted sequences, surrounded by diffuse marrow edema, which corresponds to subchondral fractures and associated reparative processes [7]. The presence of high signal intensity between the hypointense band and the articular surface on T2-weighted or contrast-enhanced images is a distinctive feature suggestive of early subchondral insufficiency fractures and is a primary element in the differentiating RPOH from idiopathic osteonecrosis of the femoral head (ONFH), where the subchondral bone is typically necrotic. MRI also frequently detects abnormalities in the acetabulum prior to the onset of joint destruction on radiographs, proving its role in the early diagnosis. MRI findings in the early stage include marked loss of articular cartilage, effusion, synovitis, and extracapsular edema. In the late stage, MRI findings typically align with those seen in radiographs, offering limited additional diagnostic value [74].

Computed tomography (CT) scans often confirm the radiological findings, indicating severe joint destruction. CT can be used to generate 3D severity maps, which provide a detailed assessment of osteoarthritic changes and acetabular anterior wall bone stock further assisting the challenges of accurate preoperative hip replacement planning [75].

## 7. Histology

RPOH synovium exhibited unique histologic features that were clearly different from those in idiopathic OA and ONFH joints, as typified by a main elevation in the infiltrated macrophages and osteoclasts [10,76]. Table 2 comprises a synthesized view on the main histological finding reported in the literature to date.

## 8. Grading Systems and Classifications

The current setting of the clinical approach on RPOH lacks a universally recognized classification system, once again highlighting the complexity of differential diagnosis and the ongoing scientific efforts to delineate its characteristics. Despite this, several grading and classification systems have been proposed in the literature, reflecting preliminary efforts to categorize this condition. We previously proposed a grading system, aiming to provide a clinico-radiological structured approach [71]. An overview of existing grading systems, as delineated in the literature, is presented in Table 3.

These classifications describe different patterns and progression rates. Irwin and Roberts identified three types of RPOH based on the duration of chondrolysis and subsequent bone loss, ranging from a rapid form with significant bone loss shortly after onset, to a delayed form where normal progression is followed by sudden deterioration [77]. Pivec et al. proposed a system based on clinical experience, differentiating between cases with rapid joint space narrowing and those with severe joint degeneration that progresses rapidly within a short time after initial symptoms [43]. In the study by Zazgyva et al., our team introduced a radiologic grading system categorizing RPOH into three grades based on joint space narrowing and the extent of femoral head deformation and ascension [71]. Later, Yasuda et al. focused on femoral head destruction, categorizing RPOH into two types: one with joint space narrowing without femoral head destruction within the first year, and another where both narrowing and destruction occur rapidly within 12 months [17]. Finally, Karayiannis et al. classified RPOH into three types, similar to Irwin and Roberts, with distinctions based on the duration of chondrolysis and the rate of bone loss [26].

The lack of a universally recognized classification system for RPOH underscores an ongoing gap in standardizing clinical diagnosis and therapy management. Existing grading systems, while valuable, reflect the complexity and variability in disease progression. Each proposed classification contributes to our understanding, yet the divergence among them underlines a need for further research to develop a cohesive, universally accepted framework. Societies worldwide should engage in promoting collaborative research establishing common guidelines and standardized criteria. Achieving this would improve diagnostic timing, accuracy, and treatment strategies.

**Table 3:** Summary of classification and grading systems for RPOH.

Reference	Publication Year	Classification	Description
Irwin LR et al. [77]	1998	Rapid	Chondrolysis for about 18 months followed by bone loss of 10–15 mm/year.
		Moderate	Chondrolysis for 18–30 months followed by bone loss at 5–10 mm/year.
		Delayed	Normal progression for 3–5 years after initial symptoms, then sudden change to rapid or moderate patterns.
Pivec R, Johnson AJ et al. [43]	2013	Type 1	Rapid joint space narrowing, no rapid femoral head dissolution or acetabular bone loss.
		Type 2	Severe joint degeneration, rapid progression with femoral head and acetabular destructive changes within 6–18 months.
Zazgyva A, Pop TS et al. [71]	2017	Grade I	Partial joint space narrowing; no deformation/ascension of the femoral head.
		Grade II	Complete disappearance of the joint space; deformed femoral head and acetabulum; femoral head ascension ≤ 0.5 cm above radiologic teardrop.
		Grade III	Complete disappearance of the joint space; partial osteolysis of the femoral head; femoral head ascension > 0.5 cm above radiologic teardrop.
Yasuda T, Hashimura T et al. [17]	2020	RPOH Type 1	Joint space narrowing with no femoral head destruction within 12 months after onset of hip pain.
		RPOH Type 2	Rapid joint space narrowing and femoral head destruction within 12 months after onset of hip pain.
Karayiannis P, Beverland D et al. [26]	2020	Type 1 (Rapid)	18 months of chondrolysis followed by 10–15 mm bone loss/year.
		Type 2 (Moderate)	18–30 months of chondrolysis followed by bone loss of 5–10 mm/year.
		Type 3 (Delayed)	Normal progression of osteoarthritis for 3–5 years before sudden deterioration into rapid or moderate types.

## 9. Discussion

This review offers a comprehensive synthesis of the current understanding of RPOH, emphasizing its pathophysiological mechanisms, risk factors, clinical presentations, imaging features, and the challenges in classification and grading systems. While many of the features of RPOH have been detailed in their respective sections, this discussion will specifically focus on the key differences between RPOH and ONFH, a disorder that is commonly misdiagnosed due to its similar clinical presentation and overlapping radiographic findings [78,79]. This comparison seeks to clarify how these conditions can be distinguished from one another, particularly in the early stages when accurate diagnosis is crucial for appropriate management.

A critical distinction between RPOH and ONFH lies in their underlying pathophysiological mechanisms. Pathologically, RPOH is commonly associated with the increased activation of the inflammasomes located in the synovium, resulting in higher levels of inflammatory markers and increased osteoclastogenesis [10]. This inflammatory response contributes to the rapid bone destruction characteristic of RPOH. ONFH typically does not exhibit these changes in the synovium. ONFH is mainly related to a reduced blood supply to the femoral head, causing bone ischemia and necrosis without the significant inflammatory process seen in RPOH [80]. Therefore, while RPOH involves an aggressive inflammatory pathway leading to rapid joint degeneration, ONFH is more associated with vascular compromise and subsequent necrosis [81]. Watanabe et al. identified that elevated serum levels of TRACP-5b, an activated osteoclast marker, are significantly higher in RPOH patients compared to those with standard OA. The ratio of TRACP-5b to total procollagen type 1 N-terminal propeptide (P1NP) was also markedly increased, suggesting that targeting osteoclast activity could suppress disease progression [82]. Yokota et al. demonstrated that markers such as NLRP3, GSDMD, IL-1β, and TNF-α are significantly elevated in RPOH, indicating a local inflammatory process that promotes osteoclastogenesis and subsequent bone destruction [10]. This contrasts with ONFH, where the primary pathology reduced blood supply, leading to bone ischemia and necrosis without significant inflammatory involvement [80]. Recent research has identified novel biomarkers that differentiate RPOH from ONFH and idiopathic OA. Floerkemeier et al. found that serum levels of adiponectin, asporin, and hypoxia-inducible factor 1-alpha can distinguish ONFH from OA with a classification accuracy of 74.5% [18]. Abe et al. highlighted that bone turnover markers like TRACP-5b and bone alkaline phosphatase are significantly elevated in RPOH compared to OA and ONFH, with TRACP-5b showing the highest diagnostic accuracy [14]. Emerging treatments targeting the specific pathophysiological mechanisms of RPOH are being explored. Given the role of increased osteoclast activity, inhibiting bone resorption presents a promising therapeutic strategy. Some authors suggest that early intervention targeting TRACP-5b could suppress disease progression. Regenerative therapies are also gaining traction. Biologics, such as platelet-rich plasma stem/stromal cells, and hyaluronic acid, are being selectively implemented based on lesion size and location, potentially delaying or reducing the need for hip arthroplasty [83]. Novel pharmacological agents, including liposome-based dexamethasone, microsphere-based triamcinolone, nerve growth factor antagonists, and anti-ADAMTS-5, aim to modify symptoms and diminish disease progression [84]. These findings prove once more the potential of serum biomarkers in improving diagnostic precision and treatment development opportunities.

Both conditions often manifest with acute hip pain and progressive joint dysfunction, and their early radiographic findings can overlap, making differential diagnosis particularly challenging in the initial stages [82]. Despite minimal radiographic abnormalities early on, patients with RPOH typically present with intense pain that rapidly progresses in a timeframe of 2–6 months [71]. Joint function usually deteriorates swiftly, with sudden joint space narrowing and subchondral bone loss that leads to femoral head destruction and resorption, but often without significant osteophyte formation. In the early phases of RPOH, patients often retain a normal range of motion during passive clinical examinations but experience severe pain during active, weight-bearing movements. This discrepancy between physical function and pain is a distinguishing feature of the condition. This clinical presentation is more prevalent in elderly women, suggesting hormonal influences on the rapid progression of the disease. Regarding clinical findings and distinguishing features, patients with RPOH typically exhibit an abrupt onset and rapid progression of severe hip pain and disability, often developing within a few months to a year. This is in contrast to the slower progression typically seen in idiopathic OA. The average duration of “symptom to surgery” in RPOH is about 1.4 years, with complete femoral head disappearance visible on radiographs within approximately 18 months [19,71]. In comparison, ONFH tends to have a more gradual onset of pain, which can be either mechanical or inflammatory in nature, and generally takes a longer time before significant joint destruction is observed [73,85]. Imaging plays an important role in differentiating RPOH from ONFH, especially in early stages where clinical presentations overlap. Key radiographic differences include an increased Tönnis angle, higher acetabular extrusion index, and abnormal lateral center-edge angle in RPOH cases [81]. On MRI, subchondral fractures, which often accompany RPOH, show a distinct low signal intensity band near the articular surface, unlike the serpiginous line typically seen in ONFH [74]. Several risk factors have been identified to play a role in the onset, development, and acceleration of RPOH. Postmenopausal women are disproportionately affected by RPOH, likely due to hormonal changes that negatively impact bone density and cartilage health [86]. Obesity, while a well-researched risk factor in osteoarthritis, has a debated role in RPOH. The use of intra-articular corticosteroids remains controversial, with some evidence suggesting they may exacerbate pre-existing joint degeneration toward RPOH [29,87]. The most commonly used steroids in these cases were triamcinolone and methylprednisolone. A review of imaging before and after injections identified that patients are generally older and have a shorter duration of symptoms before receiving an injection. Older age was found to be a significant factor associated with a higher likelihood of developing RPOH after steroid injections [73]. However, the precise relationship between intra-articular corticosteroid use, onset, and disease progression is still unclear in the current literature [32]. Classifying RPOH cases is still a challenge due to the absence of a universally recognized system. As stated previously, our team developed a clinico-radiological grading system that focused on joint space width, femoral head deformation and ascension, and onset of clinical symptoms, but was limited in terms of specific data related to ROM and laterality.

While the understanding of RPOH has advanced, particularly in its differentiation from conditions, several areas still require additional exploration. The role of angiogenesis in the progression of destructive hip osteoarthritis, especially the differences in vasculogenic profiles necessitates deeper investigation [88]. Understanding these pathways may lead to the development of treatments focused on modulating vascular factors. Research into the genetic and epigenetic changes involved in RPOH evolution, such as the differential expression of DNA methylation enzymes and PPARγ found in primary OA, could identify new molecular targets for personalized interventions [77]. These findings underscore the accelerated, rapid, and fulminant joint degeneration characteristics of RPOH, distinguishing it from other forms of OA and osteonecrosis.

Future research should focus on targeting key pathways, such as inhibiting inflammation through inflammasome blockers or reducing cartilage breakdown by inhibiting MMP-3 and MMP-9. Randomized controlled trials could assess the efficacy of these inhibitors in slowing disease progression. Additionally, longitudinal studies examining the role of genetic mutations, such as COL2A1, and pelvic misalignment could identify patients at higher risk and guide early intervention strategies.

## 10. Conclusions

This literature review highlights the complexity of RPOH, which remains a clinical challenge in diagnosis and differential diagnosis. The aggressive nature of RPOH underscores the need for further research into its causes, risk factors, and treatments. Future studies should focus on the following areas:

(a) Cellular and molecular pathways, including inflammasomes, matrix metalloproteinases, and cytokines, to identify biomarkers for early diagnosis and targeted therapies;
(b) Genetic, lifestyle, and other modifiable risk factors through large scale, long-term studies to better define the risk profile and develop preventive measures;
(c) A standardized grading system to enhance diagnosis, guide treatment options, and improve outcome assessments.

## Figures and Tables

**Table 1:** Modifiable and non-modifiable risk factors for RPOH [20,21,22].

Modifiable Factors	Non-Modifiable Factors
Regular use of NSAIDs	Advanced age
Obesity	Gender (female)
Absence of treatment for inflammatory OA	Family history
Intraarticular corticosteroids	Long-term hemodialysis (diabetic nephropathy) Sagittal spinopelvic misalignment Collagen gene mutations (COL2A1) Inflammatory pathways disruptions Cartilage metabolism disruptions

**Table 2:** Histological reported findings in RPOH [10,76].

Gross Examination	Microscopic Examination	Synovium Findings
Articular cartilage – complete cartilage loss	– bone destruction – necrotic bone fragments in a fibrotic or necrotic background	– thickening present
Subchondral bone plate – frequently associated with cartilage loss	– bone remodeling – disorganized with a hazardous pattern of trabecular bone – bone resorption – increased osteoclast activity	– fibrous tissue proliferation – aggregates of lymphocytes – bone/cartilage debris
Femoral head deformation – misshapenness and/or collapsed articular surfaces	– granuloma-like aggregates – dead bone fragments surrounded by histiocytes and osteoclastic giant cells	– inflammation absent
	– bone marrow changes – fibromyxoid changes – bone destruction – absent acute/chronic inflammation	
Cystic lesions – round, singular/multiple lesions	– fibromyxoid change in the marrow – aggregates of necrotic bone fragments/granuloma-like aggregates – increased numbers of osteoclasts	– N/A

N/A—not available.
